# School Factors Associated With the Percentage of Students Who Walk or Bike to School, School Health Policies and Practices Study, 2014

**DOI:** 10.5888/pcd13.150573

**Published:** 2016-05-12

**Authors:** Sherry Everett Jones, Sarah Sliwa

**Affiliations:** Author Affiliation: Sarah Sliwa, School Health Branch, Division of Population Health, Centers for Disease Control and Prevention, Atlanta, Georgia.

## Abstract

**Introduction:**

Active school transport, such as by walking or biking, increases physical activity levels, which has health and academic benefits for children. We examined school demographic and other characteristics to determine their association with the percentage of students who walk or bike to school.

**Methods:**

We analyzed data from the Centers for Disease Control and Prevention’s 2014 School Health Policies and Practices Study. The response rate for the module containing questions about transportation was 70% (N = 577). Multivariate logistic regression models examined whether certain school characteristics were associated with a school having 26% or more of students who walk or bike to school in the morning on an average school day.

**Results:**

In most (61.5%) schools, 10% or fewer students walked or biked to school in the morning on an average school day; in 22.7% of schools, 26% or more students did so. Although having crossing guards (adjusted odds ratio [AOR] = 3.3; 95% confidence interval [CI], 1.9–6.0), having bicycle racks (AOR = 2.7; 95% CI, 1.2–5.8), and providing promotional materials to students or families on walking or biking to school (AOR = 2.9; 95% CI, 1.7–5.1) were associated with having 26% or more students who walk or bike to school, only 47.7% of schools had crossing guards, 62.4% had bicycle racks, and 33.3% provided promotional materials.

**Conclusion:**

Several low-cost or no-cost strategies were associated with having 26% or more students who walked or biked to school, but these strategies are not commonly used in schools.

## Introduction

Active transport to school, such as walking or biking, increases physical activity levels in children (1,2), and physical activity has health (1) and academic (3–5) benefits; however, the percentage of students who walk or bike to school has declined in recent decades (6,7). Concerns about time or convenience, distance from home to school, weather, and safety (related to traffic and crime) are common barriers to active school transport (8–12). Estimates vary, but studies generally find that fewer than 20% of students walk or bike to school (7,8,10). Factors that have been shown to support active school transport are the location of schools near students’ homes as well as infrastructure and policies that address safety support (12–14). Historically, schools were sited near the families they served (15), but that practice has declined: in 1969, slightly more than half of students lived a mile or more from their schools; in 2001, three-quarters did (16).

Recognizing the benefits of active school transport, the 2015 campaign “Step it up! The Surgeon General’s Call to Action to Promote Walking and Walkable Communities” encourages walking to school through community-wide approaches that address safety concerns (17). In addition, *Healthy People 2020* includes 2 developmental objectives focusing on students walking and biking to school (18). Strategies meant to promote active transportation are not well evaluated (1). Studies that try to quantify the benefits of school and environmental policies have limited generalizability because of the specific populations or regions studied (1–3,11–13,19). Nationally representative data that describe school demographic characteristics and the impact of active transportation policies on improving active transportation rates are lacking. Nonetheless, strategies promoted to improve active school transport exist; they include adopting a safe-routes-to-school program, building schools near where students live, providing bicycle racks, implementing walking school buses (in which students walk in groups), and protecting students from crime and traffic hazards. Safety can be improved, for example, by building sidewalks or reducing motor vehicle speed (eg, through reduced speed limits around school zones, traffic-calming devices, and law enforcement presence) (1,11,12,20). The objective of this study was to examine whether school demographic and other school characteristics cited in the literature as promoting active school transport were associated with the percentage of students who walk or bike to school in the morning on an average school day.

## Methods

The School Health Policies and Practices Study (SHPPS) is a national survey conducted periodically by the Centers for Disease Control and Prevention (CDC) to assess school health policies and practices at the state, district, school, and classroom levels. SHPPS 2014, conducted from February through June 2014, examined 10 components of school health in the Whole School, Whole Community, Whole Child model (21). We examined school-level data from the Healthy and Safe School Environment questionnaire. SHPPS 2014 was reviewed by the institutional review boards at CDC and ICF International, the contractor that conducted fieldwork for SHPPS 2014, and was determined to be exempt.

### Sample and survey administration

Details of SHPPS 2014 methods are described elsewhere (22). Briefly, all SHPPS 2006 questionnaires were reviewed to identify questions that did not yield useful data (eg, because of high rates of missing responses) and to identify areas of current interest not previously covered, such as factors related to school transportation. Entirely new questions and questions modified since SHPPS 2006 were cognitively tested through telephone interviews for inclusion in SHPPS 2014. Draft questionnaires were evaluated by reviewers from federal agencies, national associations, foundations, universities, and businesses nationwide.

A 2-stage sampling design was used to select a nationally representative sample of schools. In the first stage of sampling, primary sampling units (school districts or groups of school districts) were selected with probability proportional to size. In the second stage of sampling, 2 schools per level (elementary, middle, and high school) per primary sampling unit were selected. All public, state-administered, Catholic, and non-Catholic private schools with any grade kindergarten through 12 were eligible to be included in the sample. The following kinds of schools were excluded: alternative schools, schools providing services to a population of students who were also provided services at another eligible school, schools run by the Department of Defense or Bureau of Indian Education, and schools with fewer than 30 students. The sample consisted of 828 schools. The Healthy and Safe School Environment questionnaire comprised 3 modules that grouped related items so schools could identify a respondent who was responsible for or most knowledgeable about the items covered in that module. This strategy allowed for a different respondent for each module if needed. For the module on active school transportation, the response rate was 70% (577 of 828 schools); 92% of respondents self-identified as a principal, assistant principal, or other school administrator. Approximately 90% of the data was collected via computer-assisted interviews; 10% was collected through paper questionnaires.

### Study measures

SHPPS 2014 asked schools to report the percentage of students who walk or bike to school in the morning on an average school day. Response options were 10% or less, 11% to 25%, 26% to 50%, 51% to 75%, 76% to 90%, and more than 90%. Responses were dichotomized as 25% or less and 26% or more of students; this cut point was selected on the basis of the distribution of responses. The percentage of students who walk or bike to school was similar across school level, so data were aggregated. Other SHPPS 2014 questions used in this study addressed age of the school’s main instructional building and speed limit during peak school travel times (response options were 15 mph or lower, 20 mph, 25 mph, 30 mph, and 35 mph or higher and were dichotomized as ≤25 mph or ≥30 mph). For the following topics, respondents were asked to answer yes or no: whether students are prohibited from walking or biking to or from school; whether students are bused short distances to school because their walk route was deemed too hazardous (hazard busing); whether the school provides promotional materials on walking or biking to school, such as safety tips or maps of bicycle or walking routes, to students or families; whether the school has reduced speed limits during peak travel times and traffic-calming devices to slow driving speeds on the streets that abut or are adjacent to the school; and whether the school has paid or volunteer crossing guards, a walking school bus, law enforcement officials to promote traffic safety or to prevent crime near the school, or bicycle racks.

SHPPS data were linked with extant data from the Market Data Retrieval (MDR) database (http://mchdata.com). The MDR database is updated annually and contains information about individual US schools and school districts. The MDR variables included in this analysis were percentage of students eligible for free or reduced-price lunch, percentage of white students, metropolitan status, number of students enrolled in the school, and region.

The percentage of students eligible for free or reduced-price lunch ranged from 0% to 100% (mean = 50.1%; 95% confidence interval [CI], 46.2%–54.0%); these data were divided into 3 categories (0%–32%, 33%–65%, and 66%–100%) because of the nonlinear association between this variable and the percentage of students who walk or bike to school. The percentage of white students ranged from 1% to 100% (mean = 58.9%; 95% CI, 53.4–64.4); school enrollment ranged from 30 to 4,093 (mean = 487.1; 95% CI, 447.6–526.6), and age of the school’s main instructional building ranged from 1 to 163 years (mean = 47.6; 95% CI, 44.3–50.8); these were used as continuous variables. Metropolitan status was categorized as city, suburb, town, or rural. Region was categorized as West, Midwest, Northeast, and South.

### Analysis

Data were weighted to produce national estimates, and analyses were conducted using SUDAAN version 11.0.1 software (RTI International) to account for weighted data and the complex sampling design. First, logistic regression analyses examined whether each school sociodemographic characteristic was associated with having 26% or more students who walked or biked to school. Second, logistic regression analysis, adjusted for the significant sociodemographic characteristics, examined whether each activity or characteristic hypothesized to encourage active school transport was associated with having 26% or more students who walked or biked to school.

## Results

Although only a small percentage (6.5%) of schools prohibited students from walking or biking to or from school, few students actually did so. In the morning, on an average school day, 10% or fewer students walked or biked to school in 61.5% of schools, 11% to 25% of students walked or biked to school in 15.8% of schools, 26% to 50% of students walked or biked to school in 12.6% of schools, 51% to 75% of students walked or biked to school in 4.8% of schools, 76% to 90% of students walked or biked to school in 3.7% of schools, and more than 90% of students walked or biked to school in 1.6% of schools. Thus, in 22.7% of schools, 26% or more of students walked or biked to school. One in 4 (25.8%) schools used hazard busing, whereas 56.0% of schools did not use hazard busing and 18.1% of schools did not have buses.

Approximately three-quarters (76.3%) of schools had a speed limit that was 25 mph or less during peak school travel times, 68.1% had reduced speed limits on streets that abut or are adjacent to the school’s grounds during peak school travel times, 62.4% had bicycle racks, and 55.1% had law enforcement officials to prevent crime near the school (Table 1). Other strategies were found in less than half of schools.

**Table 1:** Strategies That Support Walking or Biking to School, School Health Policies and Practices Study, 2014

Strategy	Unweighted No. of Schools (%)^a^
**Speed limit during peak school travel times**	
≤25 mph	429 (76.3)
≥30 mph	139 (23.6)
**Has crossing guards**	
Yes	243 (47.7)
No	332 (52.3)
**Has walking school bus**	
Yes	32 (6.2)
No	542 (93.8)
**Has law enforcement officials to promote traffic safety near the school**	
Yes	307 (47.5)
No	267 (52.5)
**Has law enforcement officials to prevent crime near the schools**	
Yes	340 (55.1)
No	230 (44.9)
**Has bicycle racks**	
Yes	359 (62.4)
No	213 (37.6)
**Has traffic-calming devices on streets that abut or are adjacent to the school’s grounds**	
Yes	235 (40.0)
No	339 (60.0)
**Has reduced speed limits on streets that abut or are adjacent to the school’s grounds during peak school travel times**	
Yes	394 (68.1)
No	174 (31.9)
**Provides promotional materials to students or families on walking or biking to school**	
Yes	177 (33.3)
No	394 (66.7)

a Data were weighted to produce national estimates (22). Percentages may not add to 100 because of rounding. N’s do not add up to 577, because of survey participant nonresponse.

Having 26% or more students who walk or bike to school was associated with metropolitan status, region, percentage of students eligible for free or reduced-price lunch, percentage of white students, and school age (Table 2). The likelihood of having 26% or more students who walk or bike to school was significantly higher among suburban (odds ratio [OR] = 2.9; 95% CI, 1.3–6.4) and city schools (OR = 4.2; 95% CI, 1.9–9.1) than among rural schools. The likelihood of having 26% or more of students who walk or bike to school was significantly higher among schools in the West (OR = 4.3; 95% CI, 1.8–10.0), Midwest (OR = 2.9; 95% CI, 1.2–6.9), and Northeast (OR = 2.7; 95% CI, 1.0–7.0) than among schools in the South. The likelihood of having 26% or more students who walk or bike to school was significantly lower among schools in which 33% to 65% of students were eligible for free or reduced-priced lunch (OR = 0.4; 95% CI, 0.2–0.8) than among schools in which 66% to 100% of students were eligible for free or reduced-priced lunch. Having 26% or more students who walk or bike to school was negatively associated with the percentage of students who were white (OR = 0.98; 95% CI, 0.97–0.99) and the age of the school (OR = 0.99; 95% CI, 0.98–0.995).

**Table 2:** Demographic Characteristics of Schools in Which 26% or More of Students^a^ Walk or Bike to School in the Morning on an Average School Day, School Health Policies and Practices Study, 2014

Characteristic	Schools in Which 26% or More^a^ of Students Walk or Bike to School, % (95% CI)	Likelihood of Having 26% or More of Students^a^ Who Walk or Bike to School, OR (95% CI)	*P* Value^b^
**Total**	22.7 (18.3–27.8)	—	—
**School level**			
Elementary	24.1 (18.2–31.2)	1 [Reference]	—
Middle	22.1 (16.1–29.6)	0.9 (0.6–1.4)	.64
High	19.7 (14.0–27.2)	0.8 (0.5–1.3)	.31
**Metropolitan status**			
City	32.5 (23.5–43.0)	4.2 (1.9–9.1)	<.001
Suburb	25.0 (17.1–35.0)	2.9 (1.3–6.4)	.009
Town	18.5 (8.6–35.5)	2.0 (0.7–5.9)	.22
Rural	10.3 (5.7–17.7)	1 [Reference]	—
**Region**			
Northeast	24.2 (14.6–37.4)	2.7 (1.0–7.0)	.04
Midwest	25.8 (17.7–36.0)	2.9 (1.2–6.9)	.01
West	33.7 (23.9–45.2)	4.3 (1.8–10.0)	.001
South	10.6 (5.6–19.2)	1 [Reference]	—
**Percentage eligible for free or reduced-price lunch**			
0–32	26.2 (17.1–37.9)	0.6 (0.3–1.2)	.61
33–65	20.9 (14.6–29.0)	0.4 (0.2–0.8)	.008
66–100	37.2 (27.0–48.6)	1 [Reference]	—
**Percentage of white students**	—	0.98 (0.97–0.99)	<.001
**School enrollment**	—	1.00 (1.00–1.00)	.15
**School age**	—	0.99 (0.98–0.995)	.003

Abbreviations: —, not calculated; CI, confidence interval; OR, odds ratio.

a Response options were 10% or less, 11% to 25%, 26% to 50%, 51% to 75%, 76% to 90%, and more than 90%. Responses were dichotomized as 25% or less and 26% or more of students; this cut point was selected on the basis of the distribution of responses.

b *P* values determined by logistic regression

In models that adjusted for metropolitan status, region, percentage of students eligible for free or reduced-price lunch, percentage of white students, and age of the school, the percentage of students who walk or bike to school was associated with having crossing guards, having bicycle racks, and providing promotional materials to students or families on walking or biking to school (Table 3). The adjusted odds of having 26% or more students who walk or bike to school were significantly higher among schools with paid or volunteer crossing guards (adjusted OR [AOR] = 3.3; 95% CI, 1.9–6.0) than among schools without crossing guards. The adjusted odds of having 26% or more students who walk or bike to school was significantly higher among schools with bicycle racks (AOR = 2.7; 95% CI, 1.2–5.8) than among schools without bicycle racks. The adjusted odds of having 26% or more students who walk or bike to school was significantly higher among schools that provided promotional materials to students or families on walking or biking to school (AOR = 2.9; 95% CI, 1.7–5.1) than among schools that did not provide these materials. Using these 3 strategies together (crossing guards, bicycle racks, and promotional materials) may have an additive effect on active school transport. The percentage of schools with 26% or more students who walk or bike to school increased from 8.7% when none of the 3 strategies were used, to 16.0% when one was used, 28.3% when 2 strategies were used, and 42.4% when all 3 strategies were used. Some practices intended to make walking or biking safer — traffic-calming devices and reduced speed limits during peak school hours, a walking school bus, and law enforcement presence — were not associated with having 26% or more students who walk or bike to school.

**Table 3:** Schools in Which 26% or More of Students^a^ Walk or Bike to School in the Morning on an Average School Day, by Supportive Active Transportation Strategies, School Health Policies and Practices Study, 2014

Strategy	Schools in Which 26% or More of Students^a^ Walk or Bike to School, % (95% CI)	Likelihood of Having 26% or More of Students^a^ Who Walk or Bike to School, AOR^b^ (95% CI)	*P* Value
**Speed limit during peak school travel times**			
≤25 mph	24.7 (19.8–30.5)	1.4 (0.7–3.0)	.31
≥30 mph	15.9 (9.7–24.9)	1 [Reference]	
**Has crossing guards**			
Yes	32.8 (25.5–41.0)	3.3 (1.9–6.0)	<.001
No	13.5 (9.5–18.7)	1 [Reference]	
**Has walking school bus**			
Yes	39.7 (24.7–56.8)	2.2 (0.8–5.9)	.11
No	21.4 (17.0–26.6)	1 [Reference]	
**Has law enforcement officials to promote traffic safety near the school**			
Yes	23.9 (18.0–31.0)	1.2 (0.7–2.2)	.44
No	21.4 (16.3–27.4)	1 [Reference]	
**Has law enforcement officials to prevent crime near the schools**			
Yes	24.8 (19.0–31.6)	1.6 (0.9–3.0)	.11
No	20.2 (14.9–26.6)	1 [Reference]	
**Has bicycle racks**			
Yes	25.6 (19.9–32.4)	2.7 (1.2–5.8)	.01
No	17.0 (11.9–23.6)	1 [Reference]	
**Has traffic-calming devices on streets that abut or are adjacent to the school’s grounds**			
Yes	22.3 (16.0–30.3)	0.7 (0.4–1.2)	.20
No	22.7 (17.7–28.8)	1 [Reference]	
**Has reduced speed limits on streets that abut or are adjacent to the school’s grounds during peak school travel times**			
Yes	22.1 (16.9–28.3)	0.9 (0.4–1.9)	.81
No	22.3 (15.9–30.3)	1 [Reference]	
**Provides promotional materials to students or families on walking or biking to school**			
Yes	38.2 (29.9–47.2)	2.9 (1.7–5.1)	<.001
No	15.0 (11.0–20.0)	1 [Reference]	

Abbreviations: AOR, adjusted odds ratio; CI, confidence interval.

a Response options were 10% or less, 11% to 25%, 26% to 50%, 51% to 75%, 76% to 90%, and more than 90%. Responses were dichotomized as 25% or less and 26% or more of students; this cut point was selected on the basis of the distribution of responses.

b Logistic regression models adjusted for metropolitan status, region, percentage of students eligible for free or reduced-price lunch, percentage of students who are white, and age of the school.

## Discussion

This study found that for most elementary, middle, and high schools, a small proportion of students walked or biked to school in the morning on an average school day. This finding is not surprising, given that an estimated 6% of elementary, 11% of middle, and 6% of high school students could reasonably be expected to walk to school on the basis of distance and safety considerations (13) and that most studies find fewer than 20% of students walk or bike to school (7,8,10,23).

SHPPS findings that active school transport was positively associated with the percentage of students eligible for free and reduced-price lunch, negatively associated with the percentage of white students, and least common among rural schools are consistent with results from other studies among adults and children (1,23–25). Densely populated urban areas, in which racial/ethnic minority and low-income populations are overrepresented, more often have residential density and built environment features associated with walking for transportation (1,25). However, as shown in a small study of fifth-grade students in Los Angeles, social context can be of greater concern to young people than the physical aspects of walkability (11). In that study, students often avoided the shortest route to school, preferring alternate routes that had cleaner streets, no graffiti, less crime, and made walking to school feel safer or more appealing (11). Thus, although many urban environments have physical infrastructure that makes active transport more feasible than in nonurban environments, perceptions of crime-related safety among children and adults need to be addressed when promoting active transportation (8,11,12,19).

Several low-cost or no-cost strategies were associated with having 26% or more students who walked or biked to school — having paid or volunteer crossing guards, having bicycle racks, and providing promotional materials. These kinds of strategies, however, have limited impact on students whose school is located far from where they live because the distance and convenience issue is difficult to overcome (9,26) unless these strategies are combined with other promotional activities (eg, drop-off zones, that is, an off-campus location to which students can be driven and then can walk to school). The percentage of students who walk or bike to school decreases as the distance from home to school increases (6,7). Greater distances between home and school in rural communities and in the South may explain particularly low rates of active transport in those schools.

Although we did not observe an association between active commuting and traffic-calming measures, a walking school bus, or law enforcement presence, these factors are recommended by the Safe Routes to School National Partnership and the National Highway Traffic Safety Administration as a means to promote real and perceived safety near schools (20,27). Schools may not have direct control of funding for such factors as law enforcement, reduced speed limits, or traffic-calming devices. Such factors could be addressed through collaboration with local government officials or community groups (12).

This study has several limitations. First, SHPPS data are cross-sectional, so causal relationships between school practices that might encourage students to walk or bike to school and the actual percentage of students who do so cannot be determined. Indeed, schools that have greater potential for active commuting to school may be more likely to invest in strategies that increase active commuting, and as active commuting increases, more strategies may be put into place, resulting in a positive feedback loop. Second, any seasonal variation in walking or biking to school was not captured in this study. Third, we were unable to determine the distance students lived from school or the extent to which safety played a role on streets that did not abut or were not adjacent to the school grounds. Distance and safety concerns may have played a role in low walking rates. Fourth, although SHPPS procedures were designed to have the most knowledgeable respondent complete a SHPPS questionnaire or module, underreporting or overreporting may have resulted from social desirability or poor respondent knowledge.

Enabling more students to walk or bike to school will require multi-agency policy interventions that address school siting to reduce distances between schools and students’ homes (6,7,9,26) as well as walking and biking infrastructure to ensure safe commutes (20,27). SHPPS found that 25.8% of schools used hazard busing, suggesting that although some students lived near their school, walking or biking was not safe. Schools sited in residential neighborhoods, with built environment infrastructure and policies that address safety, support active school transport in several ways: they reduce travel distances to school, they reduce the need for hazard busing because neighborhood schools are less often on major arterial roads or near other hazards, they can reduce traffic congestion around schools because fewer personal vehicles are used for school transport, and they may provide convenient community access to school facilities for various purposes (12–14,28,29). SHPPS 2012 found that among districts that had initiated construction of a new school in the previous 5 years, 58% indicated that the ability to walk or bike to school was not a factor in the siting decision (30). Decisions on school siting and surrounding infrastructure, made with active transport in mind and through collaborative efforts among local government transportation, regional planning, public health, and school officials, will be most likely to improve the rate at which students to walk or bike to school (12,14).
